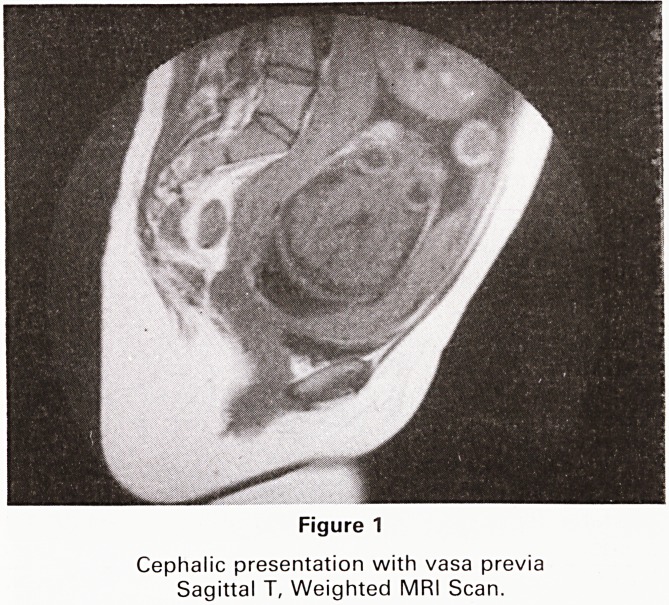# MRI in Pregnancy

**Published:** 1988-05

**Authors:** M. J. Nimmo, D Kinsella, H. S. Andrews

**Affiliations:** MB ChB


### REFERENCES

### MRI in Pregnancy: The Diagnosis of Vasa Previa by Magnetic Resonance Imaging

**M. J. Nimmo, MB ChB; D Kinsella, MRCP; H. S. Andrews, FRCR**

**Case Report**

A 24-year-old woman was admitted at 32 weeks gestation for investigation of painless antepartum haemorrhage. The pregnancy had been uneventful with a normal routine ultrasound at 16 weeks. Ultra-sound following admission demonstrated a posterior placenta within 5 cm of the internal os, and a succenturiate lobe anterioly, also within 5 cm of the internal os. The exact relationship of these structures was difficult to determine.

Following two further episodes of antepartum haemorrhage, magnetic resonance imaging was carried out at 34 weeks gestation. Sagittal and coronal sections were obtained on a Picker MRI system using T1 weighted pulse sequences (sagittal TR 300 ms, TE 30 ms, coronal TR 500 ms, TE 26 ms). Figure 1 demonstrates the normal placenta on the posterior uterine wall, terminating just over 1 cm from the internal os. A succenturiate lobe lies on the anterior wall terminating 3.5 cm from the os. Blood vessels are clearly seen between these, lying between the foetal head and the os.

**Figure 1**

Cephalic presentation with vasa previa
Sagittal T, Weighted MRI Scan.

After several further minor episodes of bleeding, an ultrasound study at 36 weeks confirmed the finding of fluid filled structures (blood vessels) between the foetal head and the os. Detailed visualisation of the placental relationship to the internal os again proved difficult.

At 36.4 weeks, a healthy boy of 2.5 kg was delivered by emergency lower section Caesarian Section. The MRI findings were confirmed at operation.

**Discussion**

The value of MRI in placenta praevia has previously been demonstrated by Powell et al (1), but no reports of vasa previa have appeared in the literature. We confirm the finding that MRI has a complementary role with ultrasound in patients where ultrasonic visualisation of the internal os and placenta is equivocal.

The high cost of MRI and its limited availability contrast with ultrasound, but ultrasound examination of patients presenting with antepartum haemorrhage will select a smaller group in which MRI will clarify equivocal appearances. In this situation its cost-effectiveness can be maximised.

### REFERENCES

1. Magnetic Resonance Imaging and Placenta Previa, Powell, M. C. Buckley, J. Price, H. Worthington, B. S. Symonds, E. M. *Am J Obstet Gynecol* 1986; **154**, 565–569.

## Figures and Tables

**Figure 1 f1:**